# Amiodarone-induced blue man syndrome: a case report

**DOI:** 10.1186/s13256-023-03954-6

**Published:** 2023-06-09

**Authors:** Ali Hossein Samadi Takaldani, Mohammad Negaresh, Maryam Salimi, Nima Javanshir

**Affiliations:** 1 Department of Internal Medicine (Pulmonology Division), School of Medicine, Ardabil University of Medical Sciences, Ardabil, Iran; 2 Department of Internal Medicine, School of Medicine, Ardabil University of Medical Sciences, Ardabil, Iran; 3 Faculty of Medicine, School of Medicine, Ardabil University of Medical Sciences, Ardabil, Iran

**Keywords:** Amiodarone, Blue man syndrome, Skin toxicity

## Abstract

**Background:**

Amiodarone is one of the most commonly used and effective antiarrhythmic drugs to treat ventricular and supraventricular arrhythmias. Besides its advantages, this drug has side effects like liver, digestive, pulmonary, thyroid, neural, skin, optical, hematologic, psychiatric, and cardiac complications. Blue-gray cutaneous discoloration, also known as blue man syndrome, is an undesirable and unusual side-effect of chronic amiodarone therapy in less than 3% of patients.

**Case presentation:**

This report presents a 51-year-old Caucasian man treated for the past 3 years with amiodarone and implantable cardioverter defibrillators due to his ventricular arrhythmia and cardiomyopathy, without any follow-up visit to his doctor. He was referred to the medical center for blue-gray discoloration on his nose and cheeks, which had started to appear in the last 3 weeks.

**Conclusion:**

Considering the findings obtained in this report and the numerous side effects of amiodarone, the blue-man syndrome is a rare yet important finding of this drug which may influence the patient’s daily life. It is recommended that all patients under treatment with this drug be notified about its side effects and visit their doctors regularly. Regarding the high therapeutic value of this drug, the lack of any association between blue man syndrome and other complications, and the related aesthetic problems, the role of the caregiver becomes much more critical in the prescription of amiodarone.

## Introduction

Amiodarone is one of the most commonly used and effective antiarrhythmic drugs to treat ventricular and supraventricular arrhythmias. However, amiodarone has the electrophysiological properties of all four classes of antiarrhythmic drugs [1]. Most of its effects are categorized under Class III. This drug exerts its antiarrhythmic effect by prolonging phase 3 of the cardiac action potential. Besides its advantages, this drug has side effects such as liver, digestive, pulmonary, thyroid, neural, skin, optical, hematologic, psychiatric, and cardiac complications [2, 3].

Blue-gray cutaneous discoloration, also called blue man syndrome, is an uncommon side-effect of amiodarone therapy [2]. It results from the accumulation of silver chemical compounds in the patient’s skin and has no proven relationship with the other side effects of amiodarone therapy [3, 4]. Blue man syndrome occurs in only 1% to 3% of patients. It is an undesirable yet benign side-effect; however, the affected patients feel stressed due to changes in the observable areas of their bodies [3]. The blue-gray discoloration of the skin is not specific to amiodarone therapy, other drugs, such as minocycline, chlorpromazine, chloroquine, and hydroxychloroquine, may also cause some level of blue-gray discoloration. Furthermore, it has also been observed in argyrosis, methemoglobinemia, and ochronosis [5].

## Case presentation

**Fig. 1:**
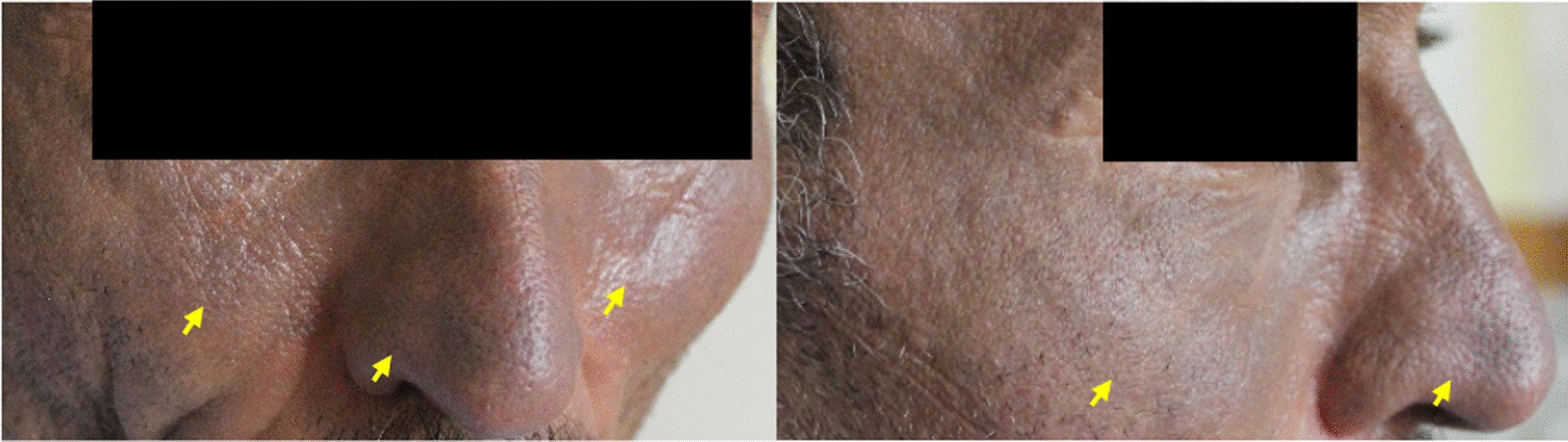
Blue man syndrome. Blue-gray discoloration on cheek and nose (yellow arrow)

The patient was a 51-year-old Caucasian man referred to the medical center with chief complaints of shortness of breath and cutaneous lesions on his cheeks and nose (Fig. 1). Shortness of breath had begun 3 years ago with the severity of modified medical research council (mMRC) II, but the severity had increased to mMRC III in the previous week, and dry coughs had also emerged. In the physical examination, a generalized wheeze was auscultated. He had no organomegaly and was not edematous.

The patient mentioned no familial or psychosocial history. He had a history of hospitalization 3 years before due to palpitation and dyspnea without chest pain. He was also diagnosed with ventricular tachycardia (VT) and ventricular fibrillation (VF). In addition, he was diagnosed with hypertension (HTN) due to a blood pressure of 180/100. His echocardiography presented an ejection fraction (EF) of 20%, severe global hypokinesia, and mild mitral regurgitation (MR). However, angiography indicated no severe coronary disease in that hospitalization. Considering the patient’s VF and the diagnosis of cardiomyopathy, implantable cardioverter defibrillators (ICD) were implanted. After being discharged from that cardiac center, amiodarone with a dose of 200 mg twice daily, furosemide tab 20 mg twice daily, spironolactone tab 25 mg twice daily, carvedilol tab 6.25 mg twice daily, losartan tab 25 mg once daily, rosuvastatin tab 10 mg once daily, and acetylsalicylic acid (ASA) tab 80 mg once daily was prescribed. Since that day, he did not refer to any medical center for follow-up and has continued using amiodarone with a daily dose of 400 mg and a cumulative dose of about 438 g. The physical examinations revealed that upon referral to the medical center, his oxygen saturation (SpO2) was 96%, blood pressure was 100/70 mmHg, body temperature was 36.5, and heart rate was 80 bpm. The patient reported smoking (20 p/y) since he was 19. However, he stopped it 10 years ago.

The skin lesions had started to appear in the last 3 weeks. The lesions were in the form of blue-gray discolorations in the skin of the nose and cheeks.

**Fig. 2:**
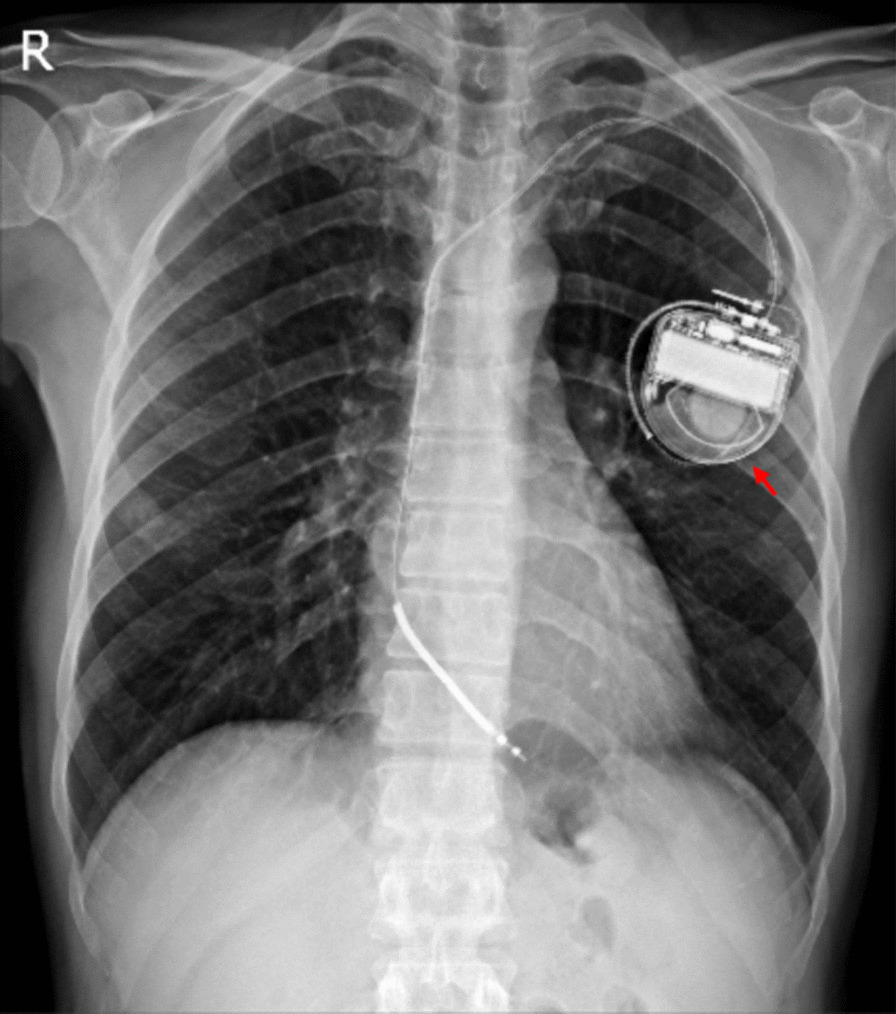
Chest imaging indicated air trapping in favor of emphysema. The red arrow shows the patient’s Implantable cardioverter defibrillators (ICD)

After admission, amiodarone was discontinued and replaced with mexiletine cap 200 mg thrice daily due to skin lesions. ICD analysis revealed a VT episode terminated by a 20 J shock that lasted 38 min. The results of thyroid function tests were normal. In the 12-lead electrocardiography (ECG) obtained from the patient, P pulmonale and left anterior hemiblock were detected. Moreover, in precordial leads, evidence of incomplete right hemiblock was observed. The serial troponin assay (× 3) and polymerase chain reaction (PCR) test for coronavirus disease 2019 (COVID-19) showed negative results. The lung images indicated air trapping (Fig. 2), and the results obtained from the pulmonary function test showed signs of obstructive lung disease, both in favor of pulmonary emphysema. The patient was discharged after a week with mexiletine cap 200 mg thrice daily, ipratropium bromide spray four puffs four times daily, and fluticasone/salmeterol 250/50 mg two puffs twice daily.

## Discussion and conclusion

This case presents a rare adverse effect of amiodarone, which, despite the cosmetic impact, does not detract from the value of amiodarone treatment. The patient mentioned no history of consuming silver-containing drugs or substances for argyrosis diagnosis [6]. Methemoglobinemia is also another differential diagnosis. It is diagnosed by the presence of cyanosis and unexplained hypoxemia in the patient. Further findings such as pallor, fatigue, headache, cyanosis, weakness, dysrhythmias, depression of the central nervous system, metabolic acidosis, seizures, coma, and death may be evident in patients with methemoglobinemia [7]. None of these symptoms were available in our patient. Also, our patient expressed no other drug usage that could cause this blue-gray discoloration.

The US Food and Drug Administration (FDA) has confirmed amiodarone for treating life-threatening and recurrent ventricular arrhythmias such as ventricular fibrillation or ventricular tachycardia. Furthermore, prospective trials have indicated that using amiodarone increases the chance of survival and improves the health status of patients suffering from myocardial infarction with left ventricular systolic dysfunction and heart failure up to class III, in terms of the New York Heart Association (NYHA) classification. Despite many favorable advantages of amiodarone, it has numerous adverse effects, of which skin reactions are one of the less dangerous side effects. The most common skin reaction is photosensitivity, with a 24%–57% prevalence among the patients. The mechanism of skin photosensitivity seems to be related to the active metabolites produced by ultraviolet rays and oxygen free radicals. After consuming a cumulative dose of at least 40 g of amiodarone, erythematous or eczematous skin lesions might emerge. They are itchy and are mainly observed in areas of the body that are exposed to sunlight, that is, hands, face, and neck. In rare cases, other side effects include alopecia, toxic epidermal necrolysis, exfoliative dermatitis, vasculitis, polyserositis, bullous dermatosis, and pustular psoriasis [2]. In amiodarone-induced skin lesions, the appearance of blue-gray discoloration is an undesirable and uncommon side-effect of this therapy [8]. The biopsy of the discolored skin indicated dense bodies attached to the lysosomal membrane, similar to lipofuscin [9]. Amiodarone might accelerate normal cellular autophagocytosis and lead to an increase in the production of lipofuscin, which is then accumulated in lysosomes. Phototoxic lesions explain the distribution of discolorations in body areas exposed to sunlight [10]. Discoloration might also result from drug deposition in the skin cells of these sunlight-receiving body parts [11].

Due to its pharmacokinetics and metabolism, oral amiodarone has a slow absorption rate with a bioavailability of about 40%. Its effects emerge a few days or weeks after starting the consumption of the drug, and the highest level of plasma is achieved after 3–7 weeks. This drug is highly lipophilic and has a long half-life (35–100 days). Bioavailability might be inhibited or intensified under the effect of age, liver disease, or interaction with other drugs. Amiodarone mainly accumulates in adipose tissue, liver, lung, and skin because of its lipophilic nature. Furthermore, it is mainly expelled via biliary excretion in the digestive system. A smaller amount of it is also discharged via urinary excretion [1, 12].

There seems to be a threshold dose for the skin side effects of amiodarone to emerge. If the daily dose is reduced to 200 mg or lower and sufficient protection against sunlight is secured, those side effects are reversible [13]. It is recommended that patients who are photosensitive or have developed blue-gray discoloration avoid exposure to sunlight, wear clothes that protect them against sunlight, and use sunscreens. Although other undesirable effects of amiodarone must be monitored and controlled, there is no relationship between them and the blue-gray cutaneous discoloration [14].

Considering the findings obtained in this report and the numerous side effects of amiodarone, the blue-man syndrome is a rare yet important finding of this drug which may influence the patient’s daily life. It is recommended that all patients under treatment with this drug be notified about its side effects and visit their doctors regularly. Regarding the high therapeutic value of this drug, the lack of any association between blue man syndrome and other complications, and the related aesthetic problems, the role of the caregiver becomes much more critical in the prescription of amiodarone.

## Data Availability

The datasets used and analyzed during the current study are available from the corresponding author upon reasonable request.

## Abbreviations

mMRC: Modified medical research council
VT: Ventricular tachycardia
VF: Ventricular fibrillation
HTN: Hypertension
EF: Ejection fraction
MR: Mitral regurgitation
ICD: Implantable cardioverter defibrillators
SpO2: Oxygen saturation
p/y: Pack/year
ECG: Electrocardiography
PCR: Polymerase chain reaction
COVID-19: Coronavirus disease 2019
FDA: The US Food and Drug Administration
NYHA: The New York Heart Association
ACC: The American College of Cardiology
AHA: American Heart Association
HRS: Heart Rhythm Society
AF: Atrial fibrillation
HF: Heart failure

## References

[1] Martino E, Bartalena L, Bogazzi F, Braverman LE (2001). The effects of amiodarone on the thyroid. Endocr Rev.

[2] Biancatelli RMC, Congedo V, Calvosa L, Ciacciarelli M, Polidoro A, Iuliano L (2019). Adverse reactions of amiodarone. J Geriatr Cardiol.

[3] Jolly U, Klein G (2016). Blue man syndrome. CMAJ.

[4] Jain SA, Rao MS, Mahesh AR. Blue in dermatology. 2022.

[5] Bracey NA, Zipursky JS, Juurlink DN (2018). Argyria caused by chronic ingestion of silver. CMAJ.

[6] Steininger H, Langer E, Stömmer P (1990). Generalized argyrosis. Dtsch Med Wochenschr.

[7] Iolascon A, Bianchi P, Andolfo I, Russo R, Barcellini W, Fermo E (2021). Recommendations for diagnosis and treatment of methemoglobinemia. Am J Hematol.

[8] Enseleit F, Wyss CA, Duru F, Noll G, Ruschitzka F (2006). The blue man: amiodarone-induced skin discoloration. Circulation.

[9] Delage C, Lagace R, Huard J (1975). Pseudocyanotic pigmentation of the skin induced by amiodarone: a light and electron microscopic study. Can Med Assoc J.

[10] Alinovi A, Reverberi C, Melissari M, Gabrielli M (1985). Cutaneous hyperpigmentation induced by amiodarone hydrochloride. J Am Acad Dermatol.

[11] Ammoury A, Michaud S, Paul C, Prost-Squarcioni C, Alvarez F, Lamant L (2008). Photodistribution of blue-gray hyperpigmentation after amiodarone treatment: molecular characterization of amiodarone in the skin. Arch Dermatol.

[12] Vyskocilova EH, Grundmann M, Duricova J, Kacirova I (2017). Therapeutic monitoring of amiodarone: pharmacokinetics and evaluation of the relationship between effect and dose/concentration. Biomed Pap Med Fac Palacky Univ Olomouc..

[13] Kounis NG, Frangides C, Papadaki PJ, Zavras GM, Goudevenos J (1996). Dose-dependent appearance and disappearance of amiodarone-induced skin pigmentation. Clin Cardiol.

[14] Epstein AE, Olshansky B, Naccarelli GV, Kennedy JI, Murphy EJ, Goldschlager N (2016). Practical management guide for clinicians who treat patients with amiodarone. Am J Med.

